# Crashworthiness of Additively Manufactured Auxetic Lattices: Repeated Impacts and Penetration Resistance

**DOI:** 10.3390/ma17010186

**Published:** 2023-12-29

**Authors:** Paolo Franzosi, Ivan Colamartino, Alessandro Giustina, Marco Anghileri, Marco Boniardi

**Affiliations:** 1 Department of Aerospace Science and Technology, Politecnico di Milano, Via La Masa 34, 20156 Milan, Italy; 2 Department of Mechanical Engineering, Politecnico di Milano, Via La Masa 1, 20156 Milan, Italy

**Keywords:** auxetic, crashworthiness, meta-materials, impact, compression, penetration

## Abstract

Auxetic materials have recently attracted interest in the field of crashworthiness thanks to their peculiar negative Poisson ratio, leading to densification under compression and potentially being the basis of superior behavior upon impact with respect to conventional cellular cores or standard solutions. However, the empirical demonstration of the applicability of auxeticity under impact is limited for most known geometries. As such, the present work strives to advance the investigation of the impact behavior of auxetic meta-materials: first by selecting and testing representative specimens, then by proceeding with an experimental and numerical study of repeated impact behavior and penetration resistance, and finally by proposing a new design of a metallic auxetic absorber optimized for additive manufacturing and targeted at high-performance crash applications.

## 1. Introduction

Auxetic materials are characterized by a negative Poisson’s ratio, responding to a compressive load with a transverse contraction: such peculiar property potentially leads to significant enhancement in terms of impact, shear, penetration, and fracture resistance. The definition was introduced in the scientific literature by Evans [1].

While auxeticity is present in nature under different forms [2], it is common today to artificially produce cellular structures [3,4,5,6], foams [7], composite materials [8,9,10], and cellular geometries with globally auxetic behavior; the latter are often called auxetic meta-materials. Among the several cellular meta-materials [11], auxetic materials have recently attracted a lot of interest in multiple fields of engineering [12]. Focusing only on studies related to impact engineering and crashworthiness in particular, as such is the field on which the present study is centred, 2D auxeticity was extensively analyzed in the literature in analytical, numerical, and experimental terms; multiple novel geometries have been designed, modeled, and produced, usually in the form of sandwich panels [13] or foam-filled tubes [14,15,16]: auxetic cores mostly consisted of planar extrusion of 2D geometries, providing useful practical demonstration of auxeticity applicability in real case studies. Such a procedure is advantageous as related core geometries are relatively easy to produce, especially with classical additive processes, while numerically, they can be treated with shell elements. On the other hand, in-plane performances of 2D cores are inevitably low with respect to the normal direction, especially in largely unstable loading conditions such as crushing impacts.

While 2D auxeticity has been comprehensively investigated by multiple authors, 3D auxetic structures have been featured less in the literature, and little experimental data can be found for what concerns impact properties. The first notable study was published in 2015 by Yang et al. [17], who experimentally and numerically investigated the mechanical properties of 3D re-entrant Honeycombs, as theorized previously by Almgren et al. [18]. Ebrahimi et al., in 2018, proposed a novel anti-chiral topology with a tunable Poisson ratio [19]. Between 2019 and 2020, Novak et al. thoroughly studied the dynamic and impact properties of metallic chiral (often addressed as "sinusoidal") structures, additively manufactured via SEBM (Selective Electron Beam Melting) and SLM (Selective Laser Melting) [20,21,22]. In 2020, Jenett et al. proposed a novel assembly mechanism targeting the mass production of large geometries [23]. Similarly, Balaji et al. presented a new production method based on the modification of the re-entrant cubic topology [24]. In 2022, Novak et al. [25] proposed a new gradable, axisymmetric chiral absorber. Recently, Varas et al. proposed a new re-entrant topology produced via SLA (stereolithography) techniques, evaluating its properties under static and dynamic compression [26]. Finally, it is worth citing the recent work of Galea et al., who proposed a novel method to produce 3D auxetic metamaterials [27].

In this context, the aim of the study was to advance the investigation of three-dimensional auxetic reticula and analyze their impact behavior with primary focus on the experimental feasibility and applicability of such structures for crashworthiness purposes. In particular, properties commonly shown in the literature as potentially present and advantageous in auxetic lattices are a peculiar triangular load curve, resistance to repeated impacts, and superior penetration resistance. However, these features, for most lattices, have not been fully investigated; the literature is lacking, especially in the experimental/numerical evaluation of low-speed repeated impacts and penetration performances, and the extension of auxetic behavior under such loading conditions. On the numerical side, it is worth noting that solid-meshed explicit models were developed, which is a strategy rarely implemented in the literature for strut-based lattices due to the difficulty in obtaining high-quality meshing and to the higher computational costs; here, such a strategy was considered necessary for the reliable construction of predictive models to be used in the penetration analyses.

First, preliminary investigations were performed on SLA-produced specimens, via static compression tests on four significant topologies, of which the two best performing were chosen to proceed with the study. Then, these two topologies were produced via SLS, and the impact properties of samples manufactured with both the technologies were evaluated. Subsequently, numerical strategies were exploited to investigate the penetration resistance. Last, the most promising topology in terms of energy absorption potential, the planar anti-chiral, was modified to allow for high-quality printing through SLM stainless steel to target applications in high-performance energy absorption.

## 2. Materials and Methods

### 2.1. Cellular Cores

Heretofore, there are several potential designs for auxetic reticula, and multiple analytical and numerical procedures have been presented for the identification and generation of novel geometries. Among such countless designs, the present study preliminary focused on four representative topologies and then proceeded with the two best-performing ones. Geometric parameters were chosen to allow for manufacturability via SLA and because they conformed to literature-proven choices; in particular, the common parameter for each topology is a strut radius of 0.5 mm.

Table 1 and Table 2 provide a brief overview of the four selected geometries together with the parameters chosen for preliminary testing and finalized choices.

In the following paragraphs, the four geometries are outlined with a comprehensive definition of their geometric parameters. Clarifying figures, inserted to provide an immediate understanding of the topological features of the structures, are mostly taken from the relevant literature, and a few annotations are added to describe the geometric parameters used. Analytical equations for relative density, a non-dimensional parameter representing the volume fraction of the material, have been computed and are reported for each topology.

#### 2.1.1. Inverted Tetrapods (ITs)

Inverted tetrapods (ITs) were initially designed by Wei et al. [28] as a micro-scale polymeric network and brought to a macro-scale lattice by Schwerdtfeger et al. [29]. It is defined by four parameters: length of the vertical strut (*h*), length of the oblique beams (*l*), angle of the overhangings from the horizontal plane (θ) and strut radius (*r*).

**Figure 1:**
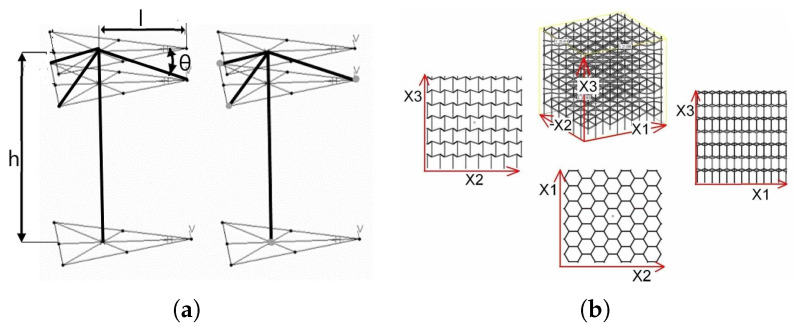
Inverted tetrapods (ITs): unit cell (**a**) and cellular structure (**b**). Pictures were taken from [29] and [30], respectively.

Figure 1 shows details of the present geometry. Inverted tetrapods are anisotropic with a negative Poisson’s ratio along the axes X1 and X2; Schwerdtfeger experimentally identified the ratio for a Ti-6Al-4V alloy produced with EBM (Electron Beam Melting), resulting in values between −0.2 and −0.4, respectively. The relative density can be calculated analytically via Equation (1), and for parameters set at *h* = 3.5 mm, *l* = 2.64 mm, θ = 11°, and *r* = 0.5 mm, the result is 0.18.

(1) $$\frac{\rho }{\rho_{0}}=\frac{8\pi r^{2}\left(h/l+3\right)}{l\left(h-l\sin\theta \right)11\sqrt{3}}$$

The unit cell and cellular parameters reported in Table 2 are defined as follows: for what concerns the unit cell dimensions, parameters refer to *h* × *l*, as defined above; the cell count in directions X1 and X2 is measured as the maximum number of complete hexagons; in direction X3, the cell count is measured as the maximum number of vertical layers; overall dimensions are intended as finalized nominal values of the specimen.

#### 2.1.2. Re-Entrant Hexagons (REHs)

**Figure 2:**
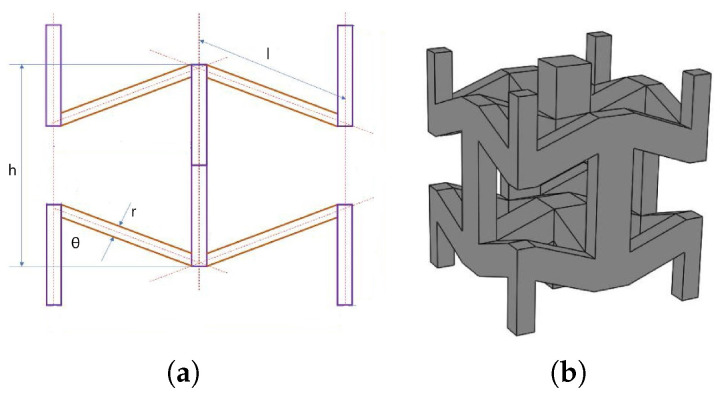
Re-entrant hexagons (REHs) lattice: 2D view (**a**) and 3D unit cell (**b**). Pictures were taken from [32].

Re-entrant hexagons (REHs) were firstly proposed by Evans et al. [7,31] in the 1990s, while comprehensive analysis of their mechanical properties was provided by Yang et al. [17]. The parameters defining this lattice are shown in Figure 2: length of the vertical struts (*h*), length of the re-entrant struts (*l*), angle of the re-entrant struts with respect to the vertical direction (θ) and radius of the struts (*r*).

With *h* = 3.75 mm, *l* = 2.58 mm, θ = 76°, and *h* = 0.5, the relative density is $\rho /\rho_{0}$ = 0.28, which is calculated through Equation (2).

(2) $$\frac{\rho }{\rho_{0}}=\frac{\pi r^{2}h/l}{2l^{2}sin\theta \left(h/l-cos\theta \right)}$$

The expected maximum Poisson’s ratio is −0.31 (Equation (3)).

(3) $$\nu_{zy}=\frac{\varepsilon_{y}}{\varepsilon_{z}}=\frac{cos\theta \left(h/l-cos\theta \right)}{sin\theta^{2}}$$

#### 2.1.3. Sinusoidal (SIN)

**Figure 3:**
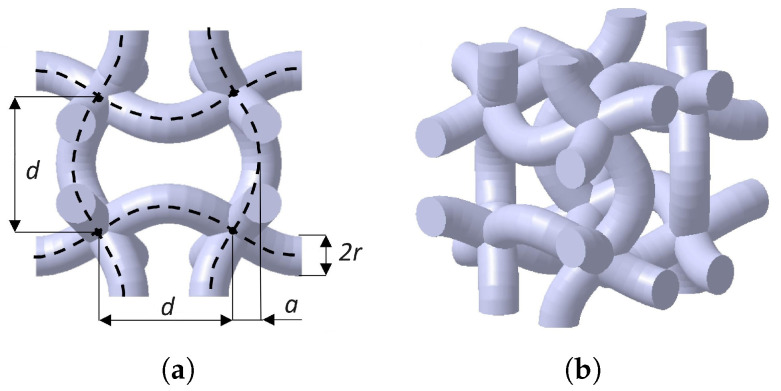
Sinusoidal (SIN) lattice: unit cell geometric parameters (**a**) and perspective view (**b**). Pictures were taken from [20].

The sinusoidal (SIN), or chiral lattice, shown in Figure 3, is based on the 10th eigenmode of the cube, three-dimensional evolution of the well-known 2D square chiral lattice structure. Among the topologies here evaluated, it is the only symmetric—and thus auxetic—in all the three directions. Hence, the Poisson’s ratio is equal in all directions and was computed at −0.4 for the geometric parameters evaluated by [33].

The parameters of the structure are the following: distance between the nodes of the cube *d*, amplitude of the sinusoidal waves *A* and radius of the strut *r*.

With parameters set as *d* = 5 mm, *A* = 1.27 mm, and *r* = 0.5 mm, the relative density of the lattice is 0.11, which is calculated as per Equation (4).

(4) $$\frac{\rho }{\rho_{0}}=\frac{12\int_{0}^{2d}\sqrt{1+{\left(A\frac{\pi }{5}sin\left(x\frac{\pi }{d}\right)\right)}^{2}}dx}{{\left(2d\right)}^{2}}$$

#### 2.1.4. Planar Anti-Chiral (PAC)

**Figure 4:**
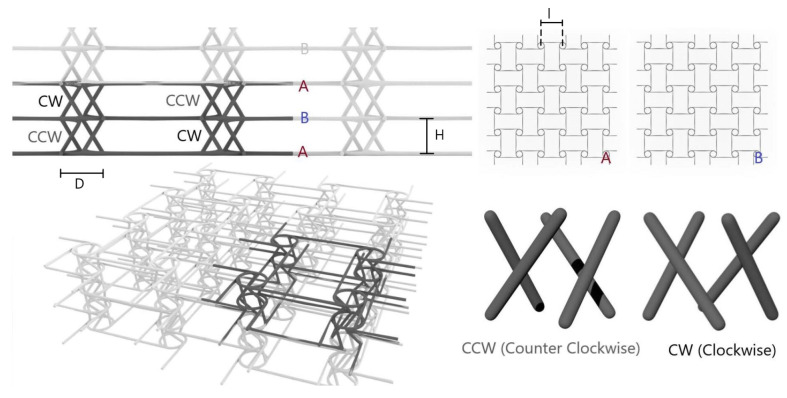
Planar anti-chiral (PAC) geometry and cellular structure.

As anticipated in Section 1, the planar anti-chiral (PAC) lattice was first proposed by Ebrahimi et al. (2018) [19]. The structure can be decomposed in two distinct parts: a planar anti-chiral geometry and a set of oblique ligaments (Figure 4): when subjected to vertical compression (that is, out-of-plane with respect to the anti-chiral plane) forces the oblique ligaments to bend, causing the rotation in two opposite direction of the planar nodes.

The parameters of the structure are ligaments length *l*, diameter of the central node *D*, distance between the planes *H* and radius of the struts *r*. With *l* = 10 mm, *D* = 6 mm, *h* = 5 mm, and *r* = 0.5 mm, chosen to obtain the lowest Poisson’s ratio, and as such, the maximum auxeticity, the relative density is computed at 0.1 from Equation (5).

(5) $$\frac{\rho }{\rho_{0}}=\frac{4\pi r^{2}\left(\sqrt{\frac{D^{2}}{2}+h^{2}}+\frac{l}{2}+\frac{\pi D}{4}\right)}{l^{2}h}$$

The Poisson’s ratio of this structure has been investigated by Ebrahimi et al. [19] and is expected to be −0.9.

### 2.2. Manufacturing

After the quick discarding of classical filament technologies, such as FDM (Fused Deposition Modeling), due to their inability to manage low resolutions, manufacturing was initially approached via SLA, in particular through a Formlabs 3L and with its proprietary material, T2K (or Tough 2000). The first manufacturing campaign targeted the initial screening of manufacturability and mechanical properties, leading to the choice of the two best-performing topologies for comprehensive impact analysis.

SLA is an additive process based on the polymerization of photo-sensitive polymers; briefly, the technology consists of a tank filled with liquid resin, that is solidified layer by layer through a laser. The process is known to guarantee high resolution and surface finish and to offer high-performance materials in terms of toughness and impact strength. These peculiarities were seen as indisputable advantages for an initial investigation, in particular related to powder-based techniques such as Selective Laser Sintering (SLS) and Multi-Jet Fusion (MJF), which are able to manage high resolutions as well but are known for high brittleness due to intrinsic porosity issues. On the other hand, one fundamental advantage of powder-based technologies is the capability of managing overhangs (shapes that are not supported or are partially supported by previous layers), which is an issue that is instead suffered by SLA. Nonetheless, it was decided to proceed with SLA and evaluate its capability to tolerate the problem.

**Figure 5:**
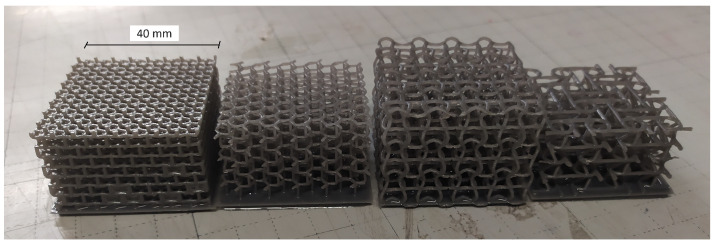
SLA samples for the preliminary quasi-static tests, left to right: re-entrant hexagons (REHs), inverted tetrapods (ITs), sinusoidal (SIN), planar anti-chiral (PAC).

Preliminary printability investigations resulted in the identification of the lower limit of a three-dimensional feature at 0.5–0.6 mm, which is a dimension that drove the design parameters and in particular the choice of *r* = 0.5 mm for all topologies. Specimens were designed through the software Grasshopper Intralattice with parameters as defined in the previous section. Overhangs had a major role in determining failure of 20–25% of the prints. A set of printed samples is shown in Figure 5.

**Figure 6:**
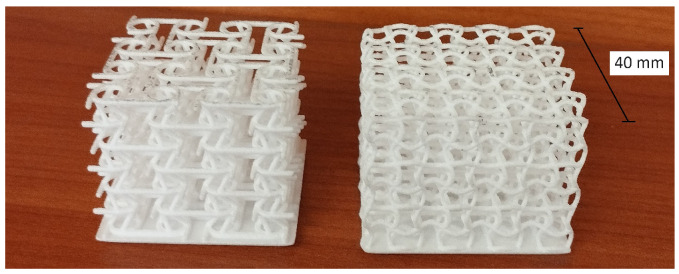
PA12 samples, left to right: planar anti-chiral (PAC) and sinusoidal (SIN).

SLA specimens production and testing resulted in two finalized geometries (the selection process and its outcomes are presented in Section 3), which were later also printed in Nylon PA12 (Figure 6) via SLS technology. After preliminary manufacturability investigations, the same geometric parameters were confirmed to be over the identified lower limit for optimal printability, reported at 0.7–0.8 mm, which is slightly higher with respect to SLA’s but nonetheless sufficiently low to allow reliable production and testing.

The two basic materials have similar mechanical properties except for elongation at break, as visible in Table 3, thus allowing for a comparison on the effect of brittleness on the lattices’ performances.

### 2.3. Experimental Methods

Experimental activities may be divided in two phases: controlled compression and impact tests.

Controlled compression was carried out through an MTS-370.10 at displacement rates of 5 mm/min, which was traceable to a quasi-static compression. The acquired signals were imposed displacement, compressive force and videos of the frontal view of the specimen.

**Figure 7:**
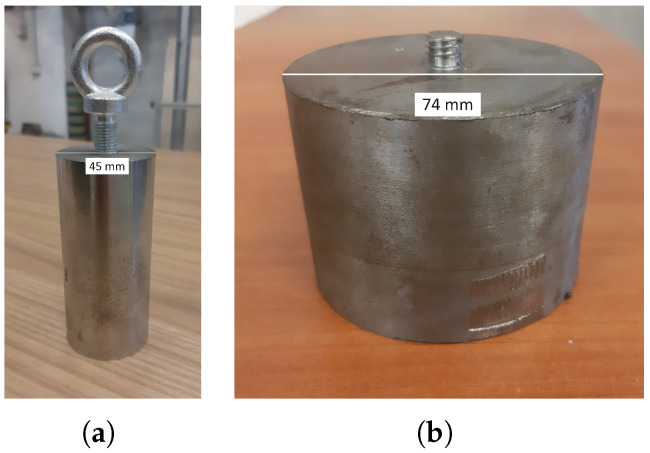
Impactors: 1.205 kg cylinder (**a**), cylindrical anvil of the 5.6 kg impactor (**b**).

Impact tests were performed internally on laboratory drop towers, loaded with a steel, cylindrical impactor of 1.205 kg weight (Figure 7) able to reach impacting energies of 0.66, 2.3 and 7 J. To distribute the impact load evenly across the impacting surface, a rigid steel plate was positioned over the specimens. Acceleration data were collected with a uniaxial piezoresistive accelerometer mounted on top of the cylinder recording at 10 kHz. The impact was further recorded by means of a high-speed camera at 10,000 fps.

A second set of repeated tests was conducted with a semi-automatic drop tower, a StepLab DW100, which was equipped with a steel cylindrical impactor of total mass of 5.6 kg (Figure 7). Data were collected through a high-performance load cell acquired at 3 MHz; an identical high-speed camera set up with respect to previous impact tests was used. The specimens were tested three consecutive times.

In the experiments, the measurement of the Poisson’s ratio was carried out assuming transverse isotropicity via the analysis of the videos. In particular, the maximum transverse contraction, always recorded at mid-height due to the friction acting on the top and bottom of the samples, was manually measured on one of the two auxetic planes. Subsequently, the ratio was calculated as per Equation (6).

(6) $$\nu_{zt}=\frac{\Delta l_{z}/L_{z}}{\Delta l_{x}/L_{x}}$$

### 2.4. Numerical Methods

Numerical models were constructed after the initial selection for both the chosen geometries, starting with simple beam-based models and scaling up with advanced material implementation including strain-rate sensitivity and a triaxiality-dependent failure model. Numerical models were developed for the Formlabs Tough 2000 material only, as proper consideration of the impact behavior of powdered materials is still today an open question in scientific research, and related implementation was beyond the scope of the work. The software used for analysis was Ansys LS-DYNA, which is the standard choice for most crashworthiness applications.

The beam models were implemented directly in LS-DYNA through Belytschko–Schwer elements with full cross-section integration. Sensitivity analyses lead to choose meshes with 20 elements per wave for the sinusoidal lattice, one element per strut and 20 per torus for the planar anti-chiral. The purpose of these models was preliminary analysis of the topologies to better design the test campaign.

Solid meshes were then implemented with two different strategies. The planar anti-chiral geometry was meshed by Python scripting through the API of Gmsh; the sinusoidal was treated with Altair Hypermesh. The resulting meshes, after sensitivity analyses, resulted in 6244 and 10,232 tetrahedral elements per cell.

The material model for the Tough 2000 was constructed and validated through dedicated tests, which is not of interest here and as such is presented in Appendix A. Finalized models exploited a piecewise linear elastic–plastic law implemented through *MAT24 with triaxiality-dependent failure to consider physically coherent struts breakage. The triaxiality curve, not available in the literature for the material under study, was taken from an internal study on a S275JR steel and linearly scaled referencing to the tensile failure strain of the material.

**Figure 8:**
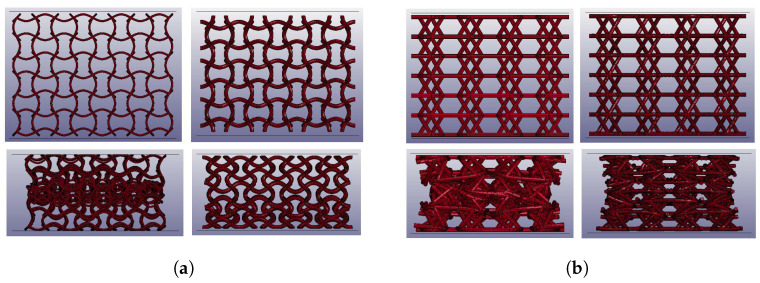
Numerical impact analyses: on the top, the meshed geometries prior to compression, on the bottom, the same geometries during compression and shortly before densification, with related contact issues visible in the beam-based models. (**a**) Sinusoidal lattice: beam (**left**) and solid meshes (**right**). (**b**) Planar anti-chiral lattice: beam (**left**) and solid meshes (**right**).

All analyses were run with the explicit scheme. For compression and impact analyses, displacement and initial velocity were, respectively, imposed to a rigid flat plate with properly adjusted mass and in contact with the lattice; the contact implemented was penalty-based and the featured friction coefficient of 0.3. As shown in Figure 8, beam-based models presented contact issues toward densification, which were successfully overcome by the adoption of solid-meshed models.

The measurement of Poisson’s ratio was carried out evaluating the maximum transverse contraction in both the auxetic directions. Subsequently, the finalized value was calculated by taking the average of the two, as per Equation (7).

(7) $$\nu_{zt}=\frac{\Delta l_{z}/L_{z}}{\frac{1}{2}\left(\frac{\Delta l_{x}}{L_{x}}+\frac{\Delta l_{y}}{L_{y}}\right)}$$

**Figure 9:**
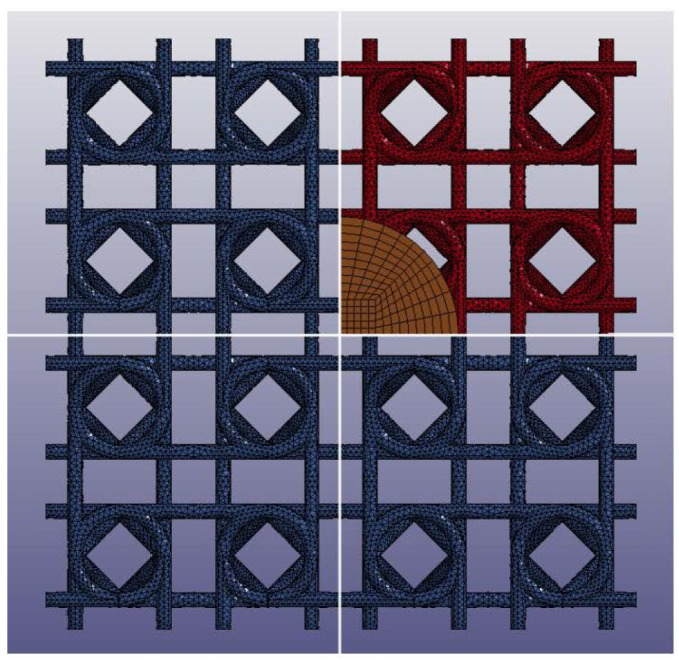
Low-speed penetration models, top view: the whole geometry (blue) and its symmetric portion (red).

For penetration, multiple solid models were constructed; then, they were reduced to a quarter exploiting symmetry, and initial velocities were imposed to the rigid impactor (Figure 9). The impactor was always centred with respect to the cellular geometry with nodal displacements perpendicular to the two symmetry planes fixed at zero. The contact implemented was, as for the impact analyses, a penalty-based contact with a friction coefficient set at 0.3.

## 3. Results

### 3.1. Quasi-Static Tests

**Figure 10:**
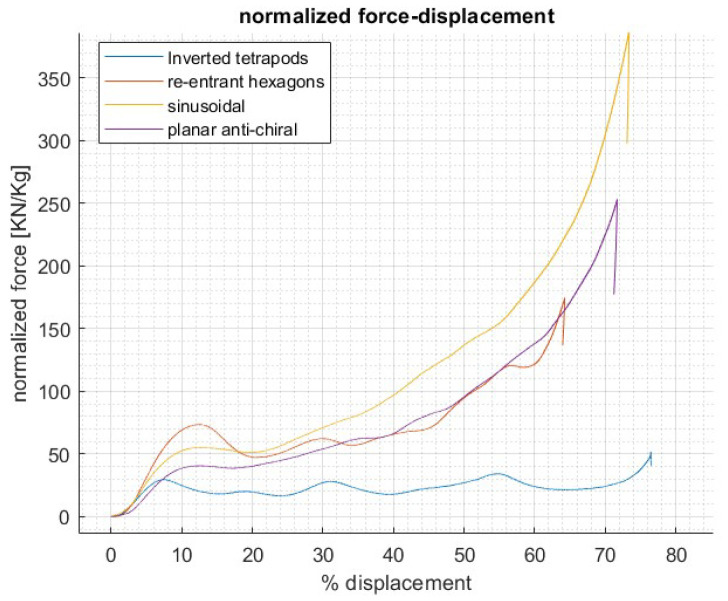
Quasi-static test results.

Static compression tests, performed with T2K resin specimens as described in Section 2.3, involved all of the four presented geometries to compare the performances of the designs (Figure 10). Since this was a preliminary test campaign, intended to lead to a first initial screening and selection of the two best-performing structures, only one sample per geometry was tested.

Stress–strain curves of the selected lattices highlighted two main results: the first is that the reaction force is increasing along the whole deformation due to the shrinking-induced interactions between the lattice beams in contrast to conventional non-auxetic lattices, which offer a plateau or a succession of peaks and valleys in the central section of the curve; the second is that toward densification, a bottoming point is not easily discernible as in conventional lattices with a gradual steepness increase. Such behavior, peculiar of auxetic structures, was however not observed for the inverted tetrapods geometry due to multiple local failures of the struts within contraction.

**Figure 11:**
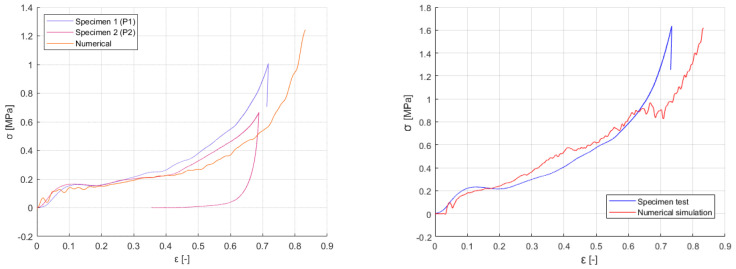
PAC (**left**) and SIN (**right**) quasi-static re-tests and numerical correlation; being the re-tests phase preliminary to the impact testing campaign, more demanding and relevant to the work, few samples were used: two for PAC, and one for the SIN lattice.

IT and REH showed a sequential failure layer by layer, originating the oscillations observable in the curve, which is behavior found also in most of the studies in the literature, while SIN and PAC reported an almost simultaneous bending of the vertical beams leading to optimal auxetic deformation. These results led to choosing PAC and SIN to proceed with the investigation. Further tests were carried out in parallel to numerical analyses, of which the final correlation is shown in Figure 11.

**Figure 12:**
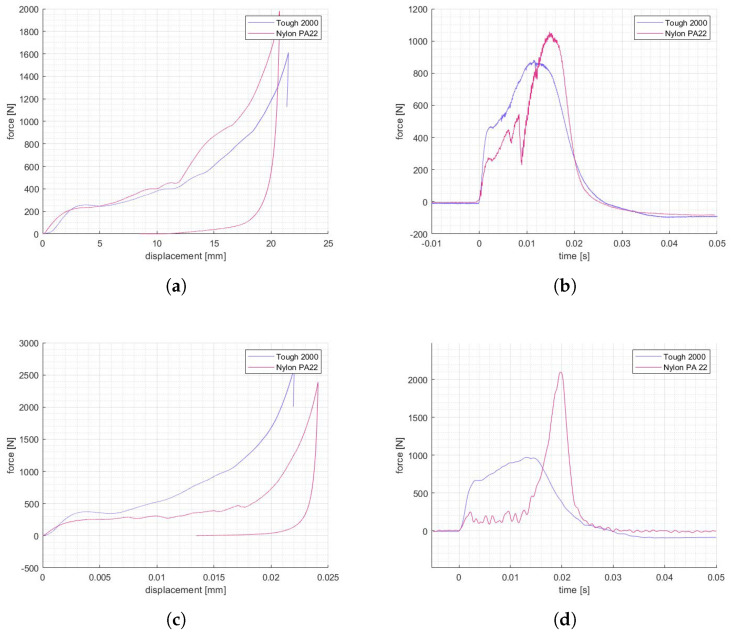
Static (**a**) and impact (**b**) compression of PAC lattices, static (**c**) and impact (**d**) compression of SIN lattices.

A set of PA12 specimen, one per each selected geometry, was then used to carry out further static tests, and properties were compared to the results of the resin ones (Table 4). The stress–strain curves for PAC were extremely coherent with the T2K behavior, while the SIN geometry showed pronounced brittleness and premature failure caused by the high deformations concentrated in the vertical beams. Full results are shown in the following section, where visual comparison of the stress–strain curves is provided between resin-based and powder-based materials, static and impact performances (Figure 12).

### 3.2. Impact Tests

Impact test results are shown in Figure 12.

**Figure 13:**
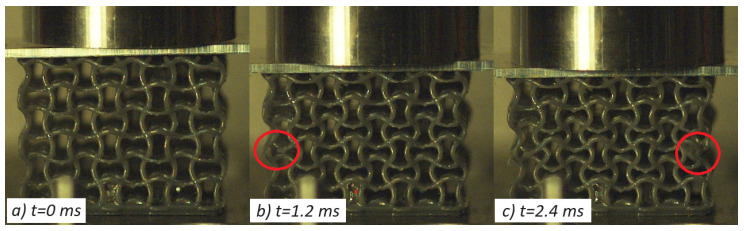
Impact test of SIN-T2K at 2.3 J with visible auxetic contraction (**c**); local failures are highlighted in frames (**b**,**c**).

**Figure 14:**
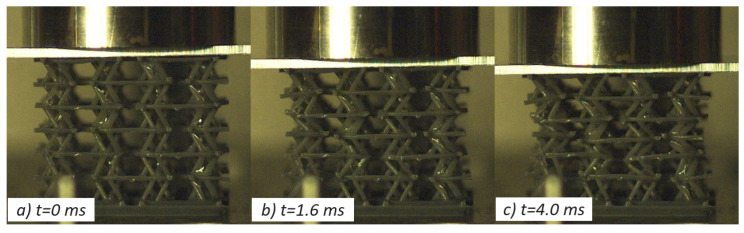
Impact test of PAC-T2K at 2.3 J, with visible auxetic contraction (**c**); no local failures were reported.

Concerning the resin-based specimens, the auxetic behavior observed under quasi-static conditions was effectively translated to low impact velocities as both the selected geometries, PAC and SIN, showed a pronounced transverse contraction under all tested speed up to 5 m/s. Considerable strength increase, close to 50%, was recorded with respect to the static counterpart in accordance with the high strain-rate dependency observed during material characterization (Appendix A). The shape of the curve, and thus the lattices behavior, was coherent with what was described in the previous section, and the peculiar force response was observed. Ultimately, the resin specimens improved performances under impact with no discernible weakening of their auxetic features. A few shots of the high-speed videos are reported in Figure 13 and Figure 14.

On the contrary, nylon cores gave radically different results with respect to the static tests: the SIN specimen underlined notable brittleness, which was further worsened by the high deformation rate, causing total failure of the structure layer by layer and complete loss of the auxetic properties; PAC geometries, instead, were able to sustain the load without complete collapse of every layer and maintaining the auxeticity with the sole exception of the layer in immediate contact with the printing base.

All the geometries, except for SIN manufactured with nylon powder, recovered a notable portion of the deformation up to 70%. Specimens were then subjected to multiple impact tests in a row to investigate the structure capability of sustaining repeated impacts. The results, at each repetition, showed progressively lower force responses in the initial elastic phase, higher slopes in the subsequent crushing phase, and higher force peaks at densification. For the nylon specimens, such a trend is the most pronounced, as highlighted by the data collected in Table 5.

**Figure 15:**
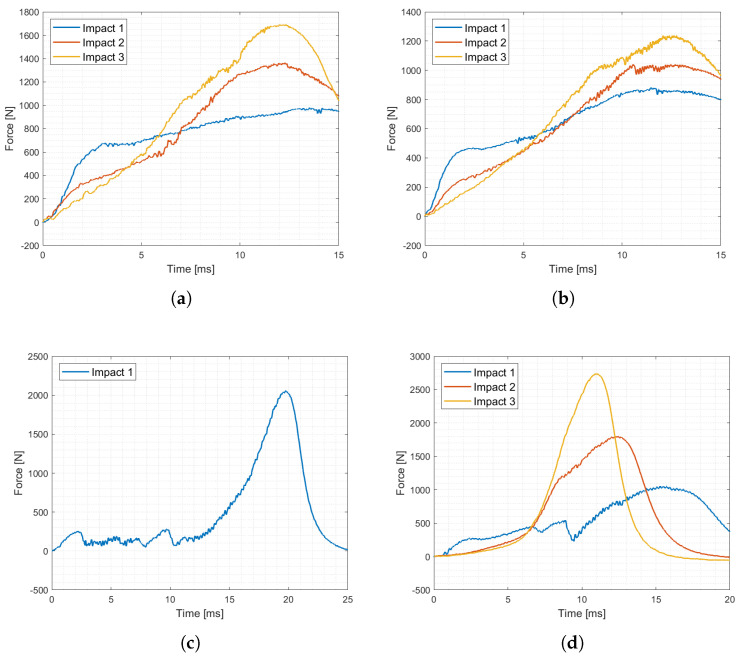
Repeated impacts results: PAC Tough 2K (**a**), SIN Tough 2K (**b**), PAC PA12 (**c**), SIN PA12 (**d**).

Of the three tested cases, the best-performing result was the PAC-T2K, able to sustain impacts with increasing peaks at an average rate of 23% per impact (Figure 15), which is in opposition to SIN-T2K and PAC-PA12, with rates of 37 and 80%, respectively.

### 3.3. Penetration Analyses

Penetration is a complex phenomenon that is intrinsically local and dependent on many factors: the shape dimension of the impactor, material properties, impact energy and speed. For a cellular material, further complication comes with the relation between the cell and impactor dimension as well as the geometric and topological configuration.

As a consequence, it was decided to study the penetration behavior as a function of significant geometric parameters for both the planar anti-chiral and the sinusoidal lattices; investigations were performed numerically as reported in Section 2.4, and for both cases, the T2K-validated resin material was used with triaxiality dependency in order to capture the material behavior up to failure. The impactor was a rigid cylinder with a hemispherical tip of radius 8 mm.

#### 3.3.1. Sinusoidal

The base sinusoidal geometry was the one reported in Section 2.1.3: from that configuration, it was decided to variate the sine amplitude, which is expressed as the percentage ratio between sine height and cell dimension and ranged between 0 and 20% with the former value equivalent to an FCC (face-centered cubic) lattice. Parallelly, the variation of impact speed was carried out with extraction of the maximum penetration depth of the impactor.

**Figure 16:**
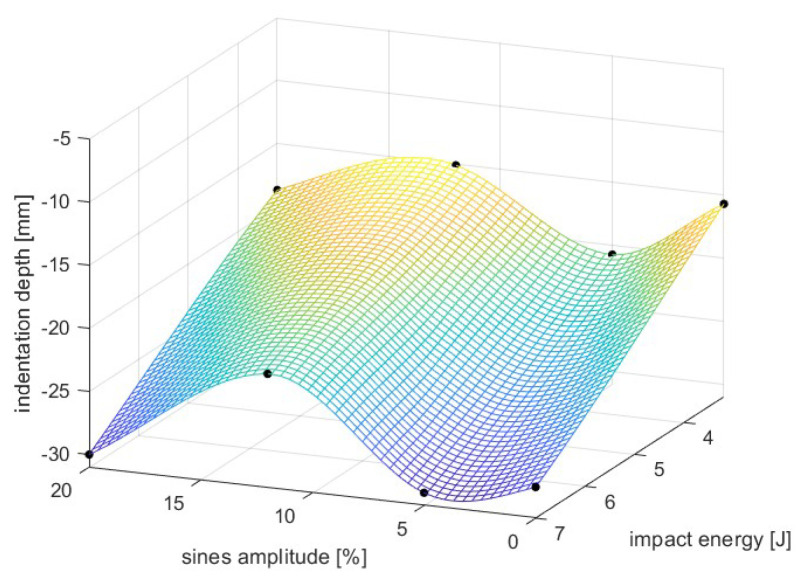
Penetration analysis of sinusoidal lattice; final results.

The results illustrated in Figure 16 reported that between amplitudes of 0% and 5%, straight (or slightly curved) beams break in bending, showing little to no auxeticity and ultimately low penetration resistance. Similar outcomes were obtained with amplitudes toward 20%: in such a case, the low clearances between the struts prevent auxetic contraction, and Poisson’s ratio, theoretically the highest, dramatically increases while penetration depths reach maximum values. The optimum was observed for amplitudes between 12 and 14%, where a compromise between large clearances and a high Poisson’s ratio allows reaching peaks of penetration resistance.

For what concerns the dependency on impact speed, an increase of penetration depth with impacting energy was reported, as reasonably expected; however, in qualitative terms, the results reported above were not affected by such energy.

Final outcomes are summarized in Figure 16.

#### 3.3.2. Planar Anti-Chiral

For the planar anti-chiral, the chosen variating parameters were the diameter of the toruses and the number of vertical layers of the lattice. The first was chosen due to its importance for the lattice mechanical properties and deformation mechanism; the second was chosen to evaluate the influence of multiple layers in the penetration response. Given that no significant dependence on the impact speed was shown for the analysis of the SIN lattices, the impact energy was kept constant at 3.2 J.

The radius of the node ranged from 2 to 3.5 mm, and each geometry iteration was tested for 3 and 6 layers of cells. As for the analyses of the sinusoidal presented in Section 3.3.1, the parameters are expressed as a percentage of the cell’s characteristic length.

**Figure 17:**
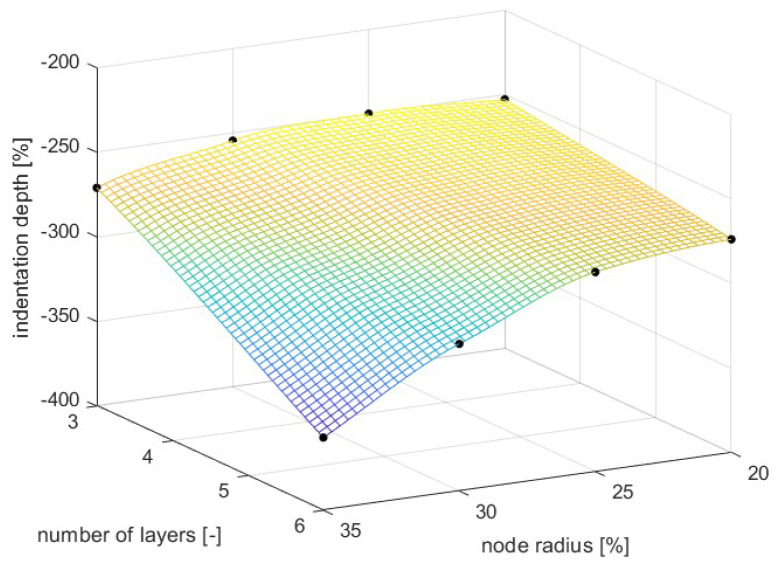
Penetration analysis of planar anti-chiral lattice, final results.

The results shown in Figure 17 indicated that the lattice performances are inversely proportional to the node radius increase. Such results were reasonably anticipated: higher radii translate in higher angles between the oblique beams and the horizontal plane, lower resisting torques of the plastic hinge at the base of the oblique ligaments and ultimately lower auxetic contraction capabilities. Since the reduction of penetration resistance is directly related to the benefits of the auxetic behavior, there was an evident decrease in the penetration resistance for low values of node radius.

For what concerns the number of layers, the six-layers geometry presented a steeper increase in penetration resistance from 35% to 20% of the radius, while the three-layer models reported a maximum at 25%; this result suggests that the optimal value of the toroidal radius is not constant as a function of the number of vertical layers but shifts backwards as the number grows.

#### 3.3.3. Non-Auxetic Geometries and Final Comparison

The unoptimized PAC and SIN were further used in a numerical comparison with non-auxetic lattices. In particular, two geometries were used: a regular BCC (Body-Centred Cubic), which was chosen as one of the most known and easily additively manufacturable geometries studied in the literature, and a stochastic Voronoi, which is surely amongst the most studied geometries in the field of mechanics of meta-materials. Geometric parameters were chosen targeting for both relative densities of 10% to allow a direct comparison in terms of penetration resistance, which is measured as the maximum vertical displacement of the impactor.

The BCC presented a cell dimension of *L* = 10 mm, and a strut radius *r* = 0.7 mm, which was set up to reach a relative density of 10%. The model was built by using Altair Hypermesh exploiting cell repetition schemes with an average element dimension of 0.5 mm.

The Voronoi, on the other hand, is a stochastic geometry and requires dedicated procedures to be properly designed and meshed for explicit analysis. For what concerns the design, parameters were chosen by exploiting recent work of the authors on stochastic Voronoi lattices [34], in particular by using the analytical equations relating relative density to cell dimension and strut diameter. Finalized meshes presented a strut radius of 0.5 mm and cell dimension of 4.7 mm. CAD geometries were automatically obtained through the related free software (https://github.com/ivncl/lattice300, 6 December 2023). Meshes were then constructed via the Altair Hypermesh Shrink Wrap feature, which is a method capable of computing high-quality meshes from complex components by approximation of the geometry; this method has recently been used by multiple researchers in the meshing of lattices [35,36] and is particularly suited for the treatment of stochastic geometries for which repetition schemes cannot be exploited [37].

Both BCC and Voronoi models were studied alongside PAC and SIN with identical setups as reported in Section 2.4. Impact energies were 3 and 7 J with lattice thicknesses set at 30 and 60 mm.

**Figure 18:**
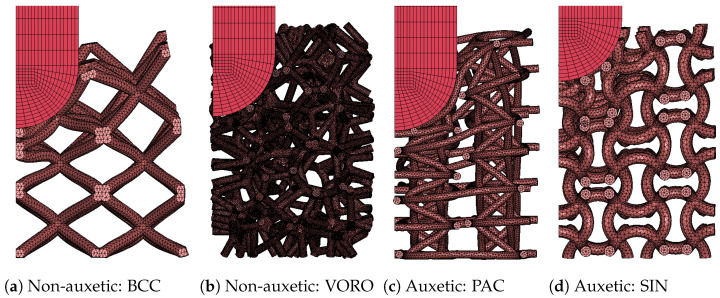
Penetration analyses at 3 J impact energy. The images capture the maximum vertical displacement of the impactor, showing the penetration resistance of the non-auxetic structures (**a**,**b**) with respect to the auxetic ones (**c**,**d**).

The results, reported in Table 6 and Figure 18, showed the best performances for the SIN lattice, followed by the Voronoi, at once confirming the benefits of the auxetic behavior for said lattice and questioning the effectiveness of the auxetic contribution for the PAC in this specific loading condition.

### 3.4. Modified Topology Investigations

After thorough analysis of polymeric lattices, it was decided to scale up the investigation in terms of absorbing performances of metallic auxetic structures and in particular additively manufactured stainless steel samples. Between the sinusoidal and the planar anti-chiral, the first has been already investigated experimentally and numerically [20,21,22], while to the authors’ knowledge, the literature still lacks proper treatment of the impact properties of the planar anti-chiral topology; furthermore, the higher crushing force carried by the sinusoidal lattice is associated with higher local stresses and lower elasticity, possibly leading to fragile rupture in impulsive conditions such as impact. Consequently, the focus of this last stage of the present research was apply the second geometry.

When manufacturing was approached, it was immediately clear that the production of metallic anti-chiral structures via SLM is problematic: the main issue was identified in the horizontal ligaments, being at the highest possible overhang with short bridging, which is a well-known complication for most additive technologies, SLM included. Even if instances of considerably overhanged cellular structures can be found in the literature, studies suggest that suboptimal results are often obtained [38], and being fundamental for the objectives of the present work analyzing practical manufacturability and design robustness, it was decided to modify the planar topology to comply to the best practices of industrial manufacturing.

**Figure 19:**
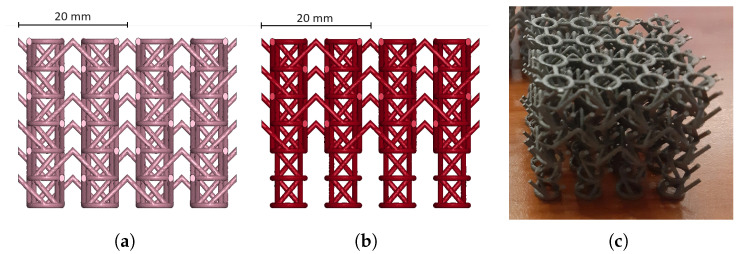
Modified planar topology, original design (**a**), defected geometry (**b**), printed samples (**c**); as easily seen in (**b**), the printed samples and numerical models are characterized by missing 45° ligaments on the whole first layer.

As such, horizontal ligaments were tilted 45°, as shown in Figure 19. The new design was assumed to cause lower values of Poisson ratio: in the planar topology, horizontal ligaments are in fact mainly responsible for auxetic contraction, and the design modification considerably changes the state of stress from purely compression/tension to bending, which is known to be less efficient. On the other hand, the overall force performances, mostly driven by the oblique ligaments’ plastic rotation, were not likely to change. The new design was applied to specimens with identical dimensions and geometric parameters as per Section 2.2.

**Figure 20:**
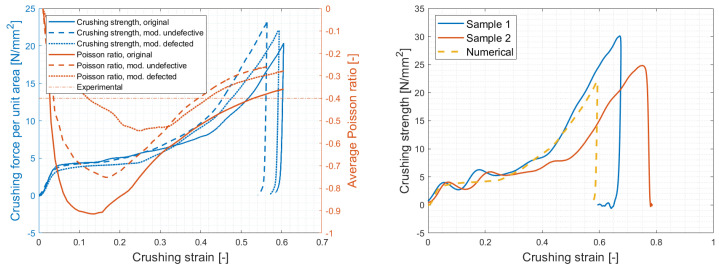
Numerical analyses of the original, modified defected and undefective geometries (**left**) and experimental results (**right**). As mentioned in the text, while the reduction of auxetic effect from original to final geometry is significant (respectively, −0.9, −0.75, −0.54, and −0.4 for original, modified undefected, modified defected and experimental result), notable differences in crushing strength performances were not identified.

Once manufactured, the specimens highlighted issues on the first of the three planar layers, resulting in immediate breakage of the V-shaped, former horizontal, ligaments (Figure 19). The defect emerged due to the immediate contact between the first printed layer and the printing plate, resulting in unsupported ligaments. Prior to testing, numerical analysis of the samples taking into account the defect were carried out, resulting in almost superimposable force signals and a notable Poisson ratio decrease, as expected due to the absence of auxeticity for 1/3rd of the samples’ height (Figure 20). These results were considered acceptable as no loss of crushing performances was evident, and proper treatment of the undefective geometry for a comprehensive overview of the absorbing properties was later addressed numerically.

**Figure 21:**
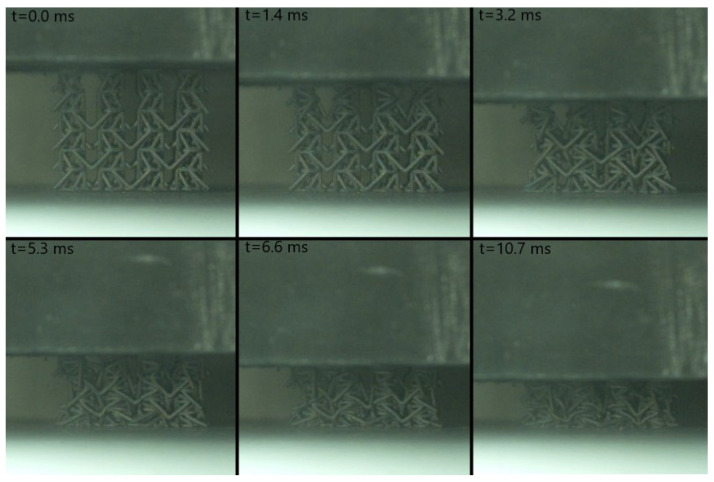
High-speed footage of the modified PAC steel specimen subjected to impact. Prior to densification, occurring at *t* = 6.6 ms, pronounced transverse contraction can be appreciated at the center of the sample.

Impact tests were performed with a drop tower equipped with 90 kg mass dropped from a height of 400 mm, resulting in 353 J of energy and an impact speed of 2.8 m/s. Acquisition consisted of the measurement of vertical acceleration history on the impactor, to extract the force–displacement compressive behavior, and high-speed videos to evaluate crushing dynamics and Poisson’s ratio evolution. Results are reported in Figure 20 and Figure 21, showing a crushing strength of 5 t/m${}^{2}$, a densification strain reported around 40–50% and specific energy absorption prior to said densification of 3.3 kJ/kg. The Poisson’s ratio was measured at −0.4 by analysis of the high-speed videos, resulting in a value that was slightly lower, in modulus, than the numerical prediction (−0.54), and considerably lower with respect to the original structure (−0.9).

As far as the crushing strength is concerned, it is worth noting the good correlation between the experimental and numerical results, which was obtained with little base material information for modeling build-up; this result suggests that the samples’ behavior had remained in the elastic and mildly plastic regions for most of the compression, proving a major auxetic contribution up to densification. On the other hand, the reason for the suboptimal Poisson’s ratio correlation can be identified in both the different measurement methods and related assumptions and the local geometric defects not implemented in the models.

**Figure 22:**
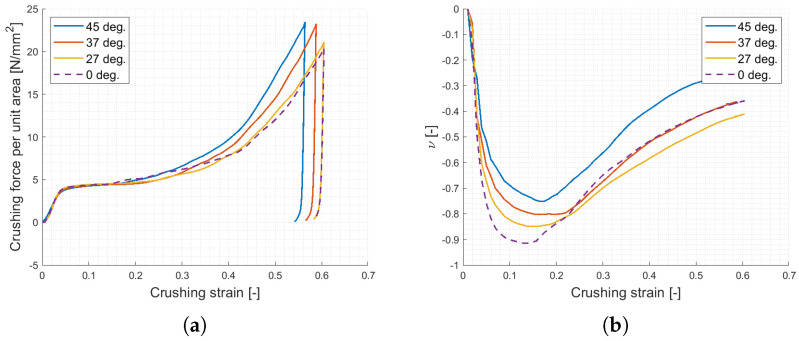
Final numerical results of the modified planar topology: crushing strength (**a**) and Poisson ratio (**b**).

Further investigations were lastly carried out to systematically consider the influence of the new design on the crushing properties. To do so, an LS-OPT routine was exploited to parametrize the topology and analyze the effects of tilting angles lower than 45 degrees. The results, shown in Figure 22, are in good agreement with the experimental signals and confirm the negligible loss of performances in terms of crushing strength with respect to the original configuration with a slight increase of the slope of the linear pre-densification phase. On the other hand, a notable loss of Poisson’s ratio was observed; in particular, the auxetic features drop to −0.75 at 45 degrees ligaments and −0.85 at 26 degrees ligaments: the trend may be approximated as parabolic, as per Equation (8).

(8) $$\nu_{zy}=\nu_{zx}=6.393*10^{-5}\;\theta^{2}+7.321*10^{-4}\;\theta -0.9147$$

## 4. Summary and Discussion

The present work aimed to study the impact properties of auxetic lattices with particular attention on an empirical demonstration of manufacturability via common additive technologies, efficacy in resisting to impact loading, and validity of auxetic behavior under dynamic crushing. The work aimed to demonstrate the benefits of auxeticity in terms of peculiar crushing performances with enhanced energy absorption, resistance to repeated impacts, superior penetration behavior, feasibility of design and the use of auxetic absorbers in high-energy applications.

The work started with the selection of four of the most commonly evaluated geometries, produced via SLA with a high-performance resin, and subsequently tested in quasi-static compression conditions. After such preliminary static testing, two geometries showing the best performances in terms of resisting force and Poisson’s ratio were probed under impact loading via single and repeated crushing. Following, the same geometries were produced via SLS and tested under similar conditions. Numerically, a preliminary study of behavior under penetration impact was carried out. Finally, an optimized geometry was constructed and produced via SLM for maximum performances; such geometry was then tested under impact with parallel comprehensive numerical analysis to robustly grasp its behavior.

### 4.1. Peculiar Crushing Behavior

**Figure 23:**
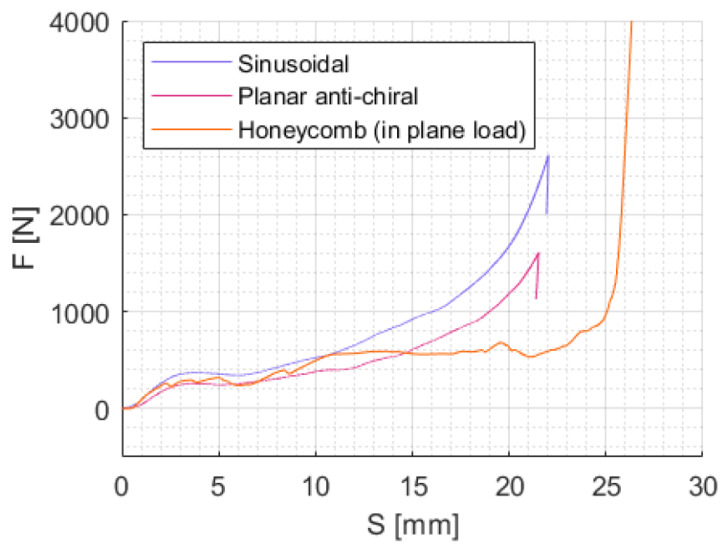
Comparison between auxetic lattices examined (sinusoidal and planar anti-chiral) and a non-auxetic honeycomb.

In the tested structures that did not exhibit elastic instability, the load–displacement curve displayed a consistent upward "triangular" trend for the load. The steepness of this curve gradually increased until total densification was achieved: the behavior is considerably different from conventional cores, which typically have a plateau either at or below their initial response level with densification leading to a sudden load peak. This characteristic is not detrimental to the crushing response; in fact, it is a significant feature in load transmission. Comparison with conventional lattices can be made in terms of the maximum load, absorbed energy and crushing stroke. Figure 23 shows the response of the two investigated lattices and a typical honeycomb core loaded in the in-plane direction, which is implemented via shell-based numerical models using the same material relative density and core dimensions, with a numerical setup identical to the one used for the static tests described in Section 2.4.

In the initial section, load levels are similar, with the honeycomb displaying lower values due to its plateau. However, at higher energy levels, the auxetic geometries show an early increase in the crushing force inevitably causing early densification though reducing the risk of bottoming phenomena.

If this unique pulse shape reduces the efficiency when compared to ideal absorbers, as expected, this peculiarity may be exploited when gradual crushing force application is needed. For example, in personal protective devices, a soft response to low-energy impacts may be desired, while a stronger response is needed for high-energy impacts. In this sense, the auxetic behavior could be an effective solution to protect from mild injuries.

### 4.2. Resistance to Repeated Impacts

When subjected to repeated tower drops, it was observed that for deformations up to 50% of the specimen’s total height, the lattice could withstand multiple impacts with only a slight increase in peak force, particularly in the case of resin-based cores. A comparison with specimens produced from nylon powders confirmed that a low brittleness of the material is key to extend auxeticity to repeated impacts. It is apparent that the elastic–plastic behavior of the core is effective only if supported by materials with a pronounced elastic/plastic range.

In this context, the planar anti-chiral geometry showed greater resistance to brittle failure when compared to the sinusoidal one with promising versatility in various applications; the sinusoidal geometries, on the other hand, work at higher global forces and local stresses, likely leading to premature local failures even for high-elongating materials. Ultimately, the core’s capability to absorb multiple impacts may be attributed to the material itself as well as to the lattice topology, and its evaluation, let alone numerical prediction, seems to require comprehensive base material characterization, including fracture and post-fracture behavior. Since such a process would be rather complex, especially for newly developed 3D-printed polymers, the authors believe that the construction of suited predictive models is necessary for the safe use of these structures.

### 4.3. Superior Penetration Behavior

Of the formulated hypotheses, the present is surely the most complicated to study let alone demonstrate in general terms due to the local effects of the phenomenon. However, numerical analyses here performed indubitably gave an affirmative response.

The results of the parametric analysis indicated that the sinusoidal cell reaches the optimum for sines amplitudes between 10 and 15% of the cell’s characteristic length in contrast to the low performances of a FCC (Face-Centered Cubic) lattice; the planar anti-chiral, instead, presented the best results for low values of toroidal node radius, and minimum penetration depth was found for a radius close to 25% of the characteristic length.

To better appreciate the penetration resistance of PAC and SIN, performances of unoptmized auxetic lattices were compared to classical cores by means of further numerical analyses of a BCC (Body-Centered Cubic) and a Voronoi lattice of similar relative density (10%). Benefits of the auxetic behavior were evident especially for the SIN lattice, while PAC reported a penetration resistance comparable to non-auxetic cores. Such a result is directly linked to the lower force carried by the PAC, which, given its higher densification strain and limited local stresses, seems to be particularly suited for resistance to repeated loading rather than to the maximization of crushing/penetration performances.

As for the repeated impacts, it is however reasonable to hypothesize that such benefits are strongly dependent on the material properties, in particular on the toughness of the T2K resin, which is able to sustain impacts without leading to local failure detrimental to the auxetic deformation. In this sense, the present results need surely to be extended not only by experimental testing on tough materials such as the T2K, but also, and most important, by developing numerical models and experimental setups able to treat and analyze the fracture behavior of 3D-printed samples, especially for powder-based materials. In parallell, the variation of geometric parameters must be carried out for both auxetic and non-auxetic geometries, targeting a comparison of optimized configurations.

### 4.4. Use in High-Performance Applications

To demonstrate the possibility of constructing high-performance, metallic, auxetic absorbers, the planar anti-chiral lattice was redesigned for metal additive manufacturability and then tested under impact.

Within crushing, the results clearly showed both pronounced auxetic behavior and high performances, with the latter not invalidated by the presence of notable manufacturing defects and reported at 5 N/mm${}^{2}$, which is comparable to high-resistance honeycombs. Furthermore, the absorber showed consistency for the two samples both in terms of qualitative deformation behavior and force performances, suggesting that the auxetic contraction is effective in reducing the probability of unsymmetrical or unstable compression. In this sense, this novel geometry, optimized for fast and reliable metal additive production, could be potentially a useful tool in conjunction with conventional absorbing structures—such as tubes—to be used as a multi-stage device able to respond to loads at different force levels. Of sure value would be further investigation with consideration of the post-impact response and analysis of its recovery capabilities.

## 5. Conclusions

The present work investigated the impact behavior of three-dimensional strut-based auxetic lattices in terms of practical manufacturability, feasibility and effective applicability for crashworthiness purposes. The activity focused at first on four representative topologies whose geometric parameters were taken from the literature; they were manufactured via resin-based additive technologies and experimentally tested under quasi-static compression. Subsequently, the two most promising geometries were thoroughly tested and modeled under impact crushing, which was followed by the evaluation of their main mechanical properties with a resin and a powder-based technology. While the powder-based samples struggled to maintain the auxetic contraction shown in quasi-static tests, the resin-based specimens reported outstanding performances and the capability of resisting multiple successive impacts. Afterwards, the response to impact penetration was studied numerically via high-quality solid-meshed explicit models, reporting high potentiality for both geometries evaluated and outperforming conventional cores. Last, the topology with higher auxetic behavior was re-designed, proposing a novel configuration optimized for metal additive manufacturing; it was then produced and tested under impact, reporting robustness and high specific crushing strength.

In conclusion, it can be surely said that, to date, the construction of effective auxetic meta-materials for impact absorption via additive techniques is a complex task; however, it is achievable. Topologies available in the literature showed particular reliability when manufactured in resin-based polymers with persistent auxeticity up to densification and the capability of sustaining repeated impact loading. Powder-based polymers presented instead multiple difficulties, demonstrating that auxetic properties are not easily transferable from static to impact compression and from one material to another: in this sense, the investigation of powder-based materials is surely needed, on both the numerical and experimental side, to design auxetic topologies suited for such technologies. However, the present work successfully demonstrated the potentiality of additively manufactured auxetic cellular structures for impact absorption, which is a step ahead of the previous literature regarding empirical demonstrations of repeated impacts and penetration behavior. Furthermore, it was shown that a robust additive production of metallic auxetic structures is achievable within standard manufacturing best practices, and high-performance devices can be obtained.

Since the present work is mostly of an empirical nature, experimental and numerical, the authors believe that future research needs to focus on the development of effective theoretical methods able to explain the repeated impact and penetration performances of auxetic lattices. Systematic numerical studies and effective experimental campaigns need to be carried out to better understand the penetration problem, targeting the reliable, generalized prediction of penetration depth as a function of geometric and material parameters. In parallel, focus must be put on the investigation of powder-based polymers with the development of robust strategies for the minimization of threatening consequences of their manufacturing technology and suited numerical models to predict their impact behavior. Further numerical and experimental studies must also be carried out to optimize the geometry of the hereby proposed metallic absorber and develop high-performant multi-stage devices.

## Figures and Tables

**Table 1:** Geometric parameters. *h*, *l*, θ, *A*, and *D*, are defined below as a function of the topologies; *r* represents the strut radius, kept at 0.5 mm for every topology; ρ/$\rho_{0}$ is the relative density, expressed as ratio between the density of the cellular solid and the one of the related base material.

Geometry	*h* (mm)	*l* (mm)	θ (deg)	*r* (mm)	*A* (mm)	*D* (mm)	ρ/$\rho_{0}$
IT	3.5	2.64	11	0.5	-	-	0.18
RH	3.75	2.58	76	0.5	-	-	0.28
SIN	-	5	-	0.5	1.75	-	0.11
PAC	5	10	90	0.5	-	6	0.10

**Table 2:** Geometric parameters for preliminary testing and finalized geometries. Except for the inverted tetrapod geometry, for which the numbers reported in the present table are treated in Section 2.1.1, all lattices are defined as cuboid-bounded repeatable shapes.

Geometry	Unit Cell Dimensions (mm)	NCells (-)	Dimensions (mm)
IT	3.5 × 2.64	7 × 9 × 6	35 × 40 × 18
REH	10 × 10 × 10	4 × 4 × 4	40 × 40 × 40
SIN (preliminary)	20 × 20 × 10	4 × 4 × 2	40 × 40 × 30
PAC (preliminary)	10 × 10 × 10	2 × 2 × 2	40 × 40 × 20
SIN (finalized)	20 × 20 × 10	4 × 4 × 2	40 × 40 × 30
PAC (finalized)	10 × 10 × 10	2 × 2 × 3	40 × 40 × 30

**Table 3:** Materials’ mechanical properties. E is Young’s modulus, $\sigma_{Y}$ is the yield stress, UTS is the ultimate tensile stress, $\varepsilon_{F}\%$ is the failure strain, ρ is the density.

Material	E (MPa)	$\sigma_{Y}$ (MPa)	UTS (MPa)	$\varepsilon_{F}$ (%)	$\rho (\mathrm{kg}/m^{3})$
Tough 2000	2000	38	40	50	1300
PA22	1500–1650	28	48	18	930
AlSi316L	180,000	530	500–600	30–40	7900

**Table 4:** Lattice mechanical properties. SEA is the specific energy absorption, E is the elastic modulus, $\sigma_{Y}$ is the collapse stress, ν is Poisson’s ratio.

-	SEA (KJ/Kg)	E (MPa)	$\sigma_{Y}$ (MPa)	ν (-)
Sinusoidal T2K	2.24	3.43	0.232	−0.37
Sinusoidal PA12	2.13	2.1	0.169	−0.3
Anti-chiral T2K	1.61	2.4	0.16	−0.81
Anti-chiral PA12	1.74	2.47	0.15	−0.83

**Table 5:** Multiple impact load peak.

-	Impact 1 (F (N))	Impact 2 (F (N))	Impact 3 (F (N))
Sinusoidal T2K	983.6	1372.2	1698.5
Sinusoidal PA12	2102.7	-	-
Planar Anti-Chiral T2K	845.5	1031.7	1238.2
Planar Anti-Chiral PA12	1057.4	1796.3	2737.6

**Table 6:** Final penetration results: maximum vertical displacement of the impactor (mm).

Lattice	30 mm–3 J	30 mm–7 J	60 mm–7 J
BCC	13.6	20.3	23.2
VORO	12.3	17.1	13.2
PAC	14.1	20.5	24.1
SIN	7.9	11.5	12.8

**Figure A1:**
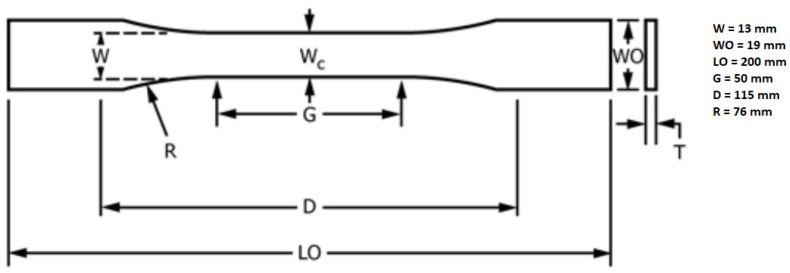
Dogbone specimens dimensions.

**Figure A2:**
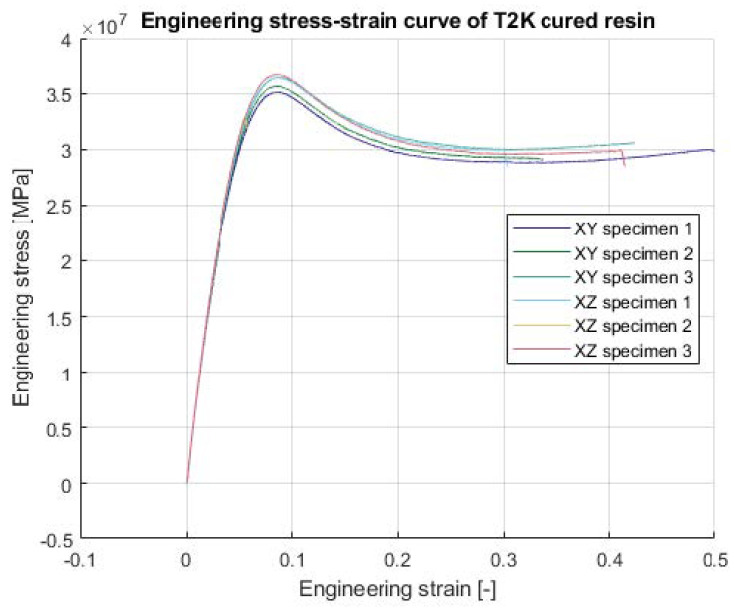
Tough 2000 tensile tests.

**Figure A3:**
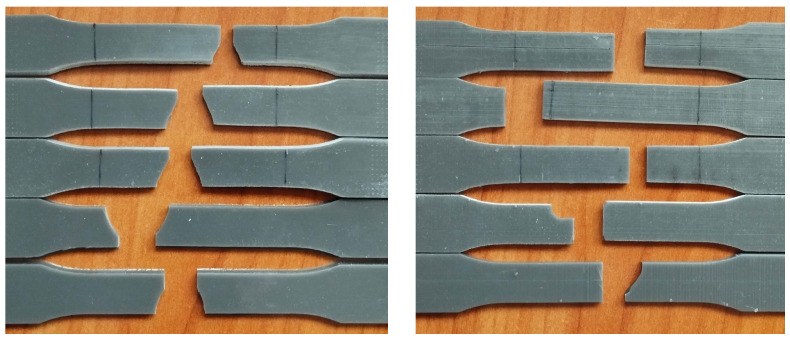
Tensile failure in XY (**left**) and XZ (**right**) direction.

**Figure A4:**
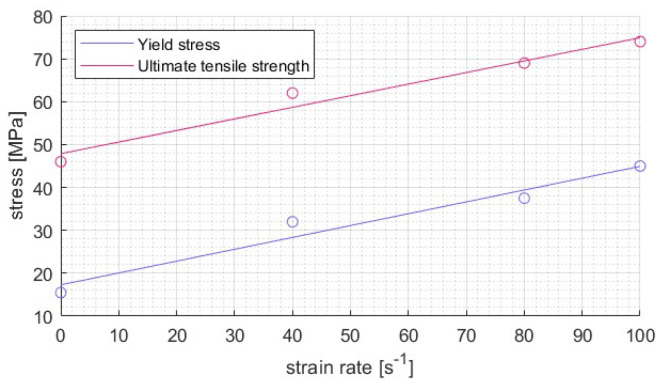
Dynamic tensile tests results.

## Data Availability

Data will be made available on request.

## Abbreviations

The following abbreviations are used in this manuscript:

API	Application Program Interface
BCC	Body-Centered Cubic
EBM	Electron Beam Melting
FDM	Fused Deposition Modeling
IT	Inverted Tetrapod
PAC	Planar Anti-Chiral
RH	Re-Entrant Hexagon
SEBM	Selective Electron Beam Melting
SIN	Sinusoidal
SLA	Stereolithography
SLM	Selective Laser Melting
SLS	Selective Laser Sintering

## Appendix A. Characterization of Formlabs Tough 2000

Since data on Formlabs Tough 2000 were not available in the literature, in particular as far as the dynamic properties are concerned, dedicated test campaigns were carried out to extract the material properties in order to properly set up the numerical models. The campaign was composed of static and dynamic tests. Specimens were classical dogbones as per ASTM D638-14 [39] for both campaigns (Figure A1).

### Appendix A.1. Quasi-Static Tests

Quasi-static tests were conducted on an MTS-370.10 machine, at speeds of 5 mm/min (0.0016 s${}^{-1}$); in total, six tests were performed at this strain rate, three specimens for each printing orientation to evaluate the degree of isotropy of the printed material. A strain gauge was also applied to each specimen to record the strain history at the expected failure point. Prior to testing, the samples were treated with dedicated painted patterns to perform DIC analyses, which were fundamental to correlate global and local strain measurement methods and calculate transverse strain and related Poisson’s ratio.

The results, as shown in Figure A2, clearly showed quasi-isotropic behavior up to failure with almost superimposable curves and considerably repeatable outcomes. Even the failure strain, calculated through the strain gauge, presented notable variability between 30% and 50% and did not exhibit dependence on the printing direction.

Failure of the material was brittle, not showing prominent necking effects, while of notable importance seemed to be the layering direction with respect to the rupture dynamics and shape, as shown in Figure A3: The XZ specimen have a neat inter-layer failure, while the XY specimen have an 45° failure line with detachment of a triangular chip; such a mechanism was coherent between the two sets.

### Appendix A.2. Dynamic Tests

Dynamics tests were aimed to investigate the strain rate dependency, obtaining the variation of the tensile properties at increasing deformation rates. Low deformation rates (40, 80, 100 s${}^{-1}$) were analyzed here, as crashworthiness properties were of interest. In order to execute the test, a Step Lab DW1000 was exploited: the lower part of the dogbone was clamped to a T-shaped grip, free to move, while the upper part was clamped to a rigid support equipped with a force sensor: in the present case, a Dytran 1210V4. The tensile dynamic load was then applied through a falling mass mounted on a vertical sled, released from the designed height, impacting the lower grip and quickly tearing the specimen.

As commonly expected from similar tests, dynamic tests reported an increasing in both yield and ultimate stress. Results are shown in Figure A4.

## References

[1] Evans K.E. (1991). Auxetic polymers: A new range of materials. Endeavour.

[2] Carneiro V.H., Meireles J., Puga H. (2013). Auxetic materials—A review. Mater. Sci..

[3] Baughman R., Shacklette J., Zakhidov A., Stafström S. (1998). Negative Poisson’s ratios as a common feature of cubic metals. Nature.

[4] Kimizuka H., Kaburaki H., Kogure Y. (2000). Mechanism for Negative Poisson Ratios over the *α*- *β* Transition of Cristobalite, *SiO*_2_: A Molecular-Dynamics Study. Phys. Rev. Lett..

[5] Grima J.N., Winczewski S., Mizzi L., Grech M.C., Cauchi R., Gatt R., Attard D., Wojciechowski K.W., Rybicki J. (2015). Tailoring Graphene to Achieve Negative Poisson’s Ratio Properties. Adv. Mater..

[6] Poźniak A.A., Wojciechowski K.W., Grima J.N., Mizzi L. (2016). Planar auxeticity from elliptic inclusions. Compos. Part Eng..

[7] Evans K., Nkansah M., Hutchinson I. (1994). Auxetic foams: Modelling negative Poisson’s ratios. Acta Metall. Mater..

[8] Milton G.W. (1992). Composite materials with poisson’s ratios close to −1. J. Mech. Phys. Solids.

[9] Wang Y. (2022). Auxetic Composite Laminates with Through-Thickness Negative Poisson Ratio for Mitigating Low Velocity Impact Damage: A Numerical Study. Materials.

[10] Lin W., Wang Y. (2023). Low velocity impact behavior of auxetic CFRP composite laminates with in-plane negative poisson’s ratio. J. Compos. Mater..

[11] Gibson L.J. (2003). Cellular solids. Mrs Bull..

[12] Novak N., Vesenjak M., Ren Z. (2016). Auxetic cellular materials—A review. Stroj. Vestn. J. Mech. Eng..

[13] Usta F., Türkmen H.S., Scarpa F. (2021). Low-velocity impact resistance of composite sandwich panels with various types of auxetic and non-auxetic core structures. Thin Walled Struct..

[14] Gao Q., Liao W.H., Huang C. (2020). Theoretical predictions of dynamic responses of cylindrical sandwich filled with auxetic structures under impact loading. Aerosp. Sci. Technol..

[15] Usta F., Ertaş O.F., Ataalp A., Türkmen H.S., Kazancı Z., Scarpa F. (2019). Impact behavior of triggered and non-triggered crash tubes with auxetic lattices. Multiscale Multidiscip. Model. Exp. Des..

[16] Günaydın K., Gülcan O., Türkmen H.S. (2023). Experimental and numerical crushing performance of crash boxes filled with re-entrant and anti-tetrachiral auxetic structures. Int. J. Crashworthiness.

[17] Yang L., Harrysson O., West H., Cormier D. (2015). Mechanical properties of 3D re-entrant honeycomb auxetic structures realized via additive manufacturing. Int. J. Solids Struct..

[18] Almgren R. (1985). An isotropic three-dimensional structure with Poisson’s ratio = −1. J. Elast..

[19] Ebrahimi H., Mousanezhad D., Nayeb-Hashemi H., Norato J., Vaziri A. (2018). 3D cellular metamaterials with planar anti-chiral topology. Mater. Des..

[20] Novak N., Starčevič L., Vesenjak M., Ren Z. (2019). Blast response study of the sandwich composite panels with 3D chiral auxetic core. Compos. Struct..

[21] Novak N., Vesenjak M., Tanaka S., Hokamoto K., Ren Z. (2020). Compressive behavior of chiral auxetic cellular structures at different strain rates. Int. J. Impact Eng..

[22] Novak N., Krstulović-Opara L., Ren Z., Vesenjak M. (2020). Compression and shear behavior of graded chiral auxetic structures. Mech. Mater..

[23] Jenett B., Cameron C., Tourlomousis F., Rubio A.P., Ochalek M., Gershenfeld N. (2020). Discretely assembled mechanical metamaterials. Sci. Adv..

[24] Balaji B., Burela R.G., Ponniah G. (2021). Mass production of re-entrant cubic auxetic structure. IOP Conference Series: Materials Science and Engineering.

[25] Novak N., Nowak M., Vesenjak M., Ren Z. (2022). Structural optimization of the novel 3D graded axisymmetric chiral auxetic structure. Phys. Status Solidi B.

[26] Varas D., Pernas-Sánchez J., Fjeldberg N., Martín-Montal J. (2023). Experimental analysis at different loading rates of 3D printed polymeric auxetic structure based on cylindrical elements. Polym. Test..

[27] Galea R., Andre Farrugia P.S., Dudek K.K., Attard D., Grima J.N., Gatt R. (2023). A novel design method to produce 3D auxetic metamaterials with continuous pores exemplified through 3D rotating auxetic systems. Mater. Des..

[28] Wei G. (1992). Negative and conventional Poisson’s ratios of polymeric networks with special microstructures. J. Chem. Phys..

[29] Schwerdtfeger J., Heinl P., Singer R.F., Körner C. (2010). Auxetic cellular structures through selective electron-beam melting. Phys. Status Solidi B.

[30] Novak N., Vesenjak M., Ren Z. (2017). Computational simulation and optimization of functionally graded auxetic structures made from inverted tetrapods. Phys. Status Solidi B.

[31] Evans K. (1991). The design of doubly curved sandwich panels with honeycomb cores. Compos. Struct..

[32] Chen J., Tao W., Pang S. (2021). Impact Testing of 3D Re-Entrant Honeycomb Polyamide Structure Using Split Hopkinson Pressure Bar. Appl. Sci..

[33] Körner C., Liebold-Ribeiro Y. (2014). A systematic approach to identify cellular auxetic materials. Smart Mater. Struct..

[34] Colamartino I., Anghileri M., Boniardi M. (2023). Investigation of the compressive properties of three-dimensional Voronoi reticula. Int. J. Solids Struct..

[35] Peto M., Aguilar-Rosas O., Ramirez-Cedillo E., Jimenez M., Hernandez A., Siller H.R. (2021). A proof of concept study of the mechanical behavior of lattice structures used to design a shoulder hemi-prosthesis. J. Eng. Sci. Med. Diagn. Ther..

[36] Daynes S., Feih S. (2022). Bio-inspired lattice structure optimisation with strain trajectory aligned trusses. Mater. Des..

[37] Giustina A., Colamartino I., Franzosi P., Anghileri M., Boniardi M. Parametric optimization of cellular materials through LS-OPT. Proceedings of the 14th European LS-DYNA Conference.

[38] Thomas D. (2009). The Development of Design Rules for Selective Laser Melting. Ph.D. Thesis.

[39] (2022). Standard Test Method for Tensile Properties of Plastics.

